# Self-Reported Changes in Premenstrual and Menstrual Symptoms among Females Taking a Novel Multinutrient Supplement: A Pilot Study

**DOI:** 10.1016/j.cdnut.2026.109511

**Published:** 2026-08-08

**Authors:** Marleigh Smith, Susanne Mitschke, Alice van der Schoot

**Affiliations:** 1 Scientific Affairs, Ditto Daily Ltd, London, United Kingdom; 2 Clinical Research, Citruslabs, Las Vegas, NV, United States

**Keywords:** premenstrual syndrome, premenstrual dysphoric disorder, menstrual cycle, dietary supplements, saffron, vitamin D, magnesium, omega-3 fatty acids, zinc, chamomile

## Abstract

**Background:**

Premenstrual and menstrual symptoms arise in response to cyclical hormonal fluctuations and encompass psychological and physical manifestations that may significantly impair quality of life. Treatment options are limited, demonstrate inconsistent efficacy, and are associated with notable adverse effects. There is growing interest in evidence-based nutritional approaches, with several placebo-controlled trials demonstrating that certain nutrient supplements significantly alleviate menstrual-cycle-related symptomatology.

**Objectives:**

This single-arm pilot study aims to evaluate self-reported changes in premenstrual and menstrual symptoms in females taking a novel multinutrient supplement containing saffron extract, chamomile extract, essential minerals (chromium, magnesium, zinc, iron), vitamins (D, B6, C), and– omega-3 (n–3) fatty acids, using advanced encapsulation technology.

**Methods:**

Forty females (mean age: 33 y) with premenstrual and menstrual symptoms consumed the supplement daily for 3 consecutive menstrual cycles. The validated Menstrual Symptom Questionnaire (MSQ) and a 5-point severity rating scale evaluated symptoms at baseline and after each menstrual cycle.

**Results:**

Overall MSQ scores decreased significantly from baseline to cycle 1 (*P* = 0.0028), cycle 2 (*P* < 0.0001), and cycle 3 (*P* < 0.0001). Overall symptom severity scores decreased by a mean 50.42% from baseline to cycle 3 (2.58 ± 0.65 to 1.28 ± 0.83, *P* < 0.0001), corresponding to a change from distracting (moderate–severe) to barely noticeable. There were significant improvements in all measured individual physical and psychological symptoms from baseline to cycle 3 (all *P* < 0.0001). By cycle 3, 88% of participants agreed that the supplement reduced symptom severity, and 82% agreed that the supplement reduced symptom duration.

**Conclusions:**

These findings suggest that this novel multinutrient supplement may be of interest as a nutritional approach to support females throughout the menstrual cycle, warranting further investigation in randomized, placebo-controlled trials.

This trial was registered on clinicaltrials.gov as NCT07267728.

## Graphical abstract

**Figure:**
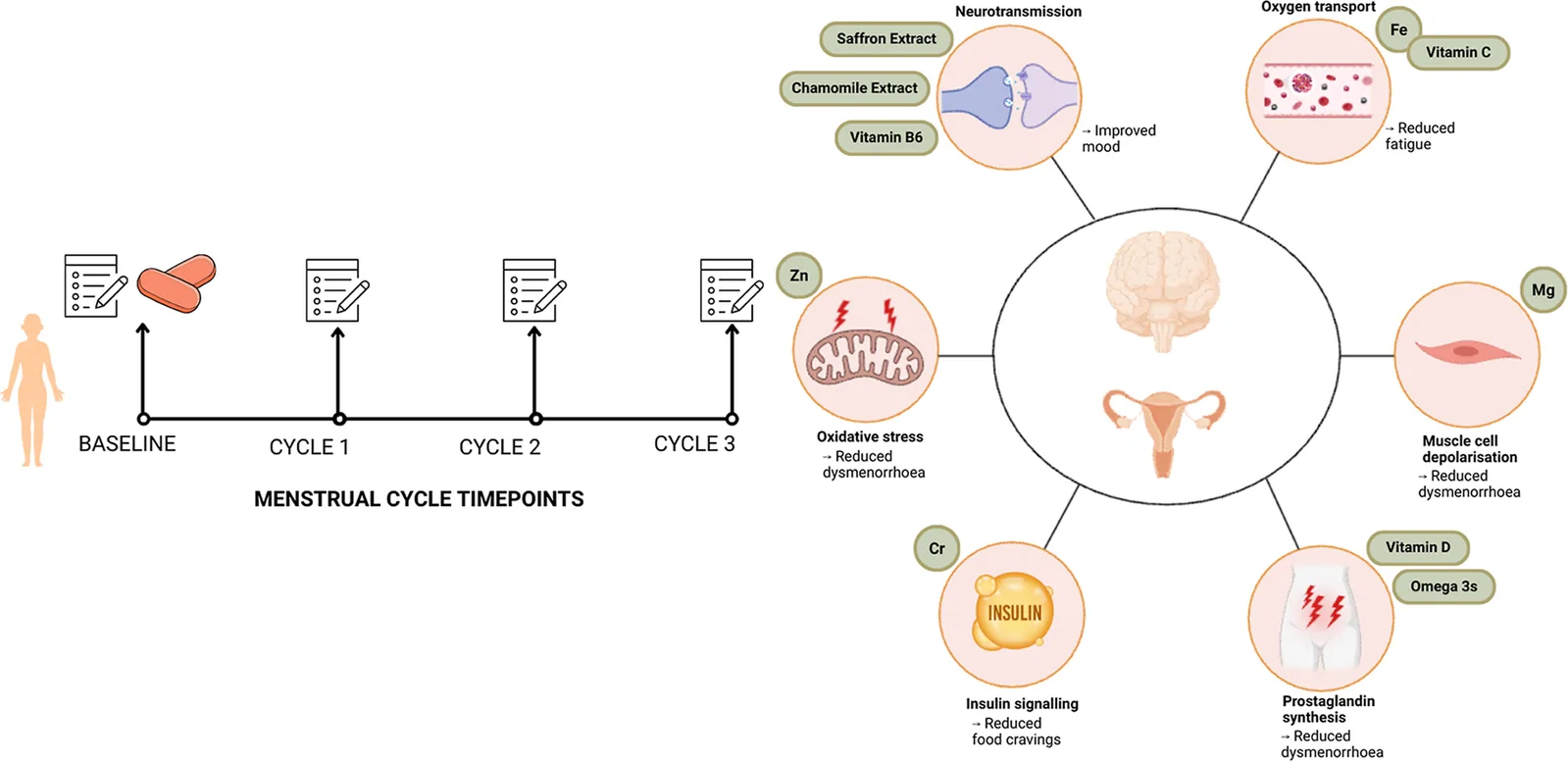

## Introduction

Menstrual cycle symptoms affect ≤90% of menstruating individuals [1]. Most commonly, these manifest as premenstrual syndrome (PMS), a spectrum of physical and psychological symptoms arising during the luteal phase of the menstrual cycle, including mood swings, irritability, cramping, bloating, breast pain, and fatigue, ranging from mild to severe [2]. For ≤8% of those with a menstrual cycle, symptoms are debilitating [3]. Premenstrual dysphoric disorder (PMDD) is a cyclical, hormone-based mood disorder that appears in the Diagnostic and Statistical Manual of Mental Disorders, 5th ed and is characterized by marked depressed mood, feelings of hopelessness, self-deprecating thoughts, anxiety, or anger that significantly impair functioning [4,5]. Beyond luteal phase symptoms, 62% of females experience severe pain during menstruation [6]. Collectively, menstrual-cycle-related symptoms can substantially reduce quality of life [4,7].

The etiopathogenesis of premenstrual disorders is not fully understood, although several pathways are implicated [[8], [9], [10], [11], [12]]. In patients with PMDD, lower γ-aminobutyric acid (GABAA) receptor δ-subunit expression and higher serotonin binding to the serotonin transporter are found in the luteal phase compared with controls, correlated with altered emotional generation through heightened amygdala activation and worse depressive symptoms, respectively [9,10]. PMS is found to involve differences in neurotransmitter functioning, inflammation, and oxidative stress, which may contribute to its severity [8,11,12]. Severe menstrual pain is associated with 4-fold higher prostaglandin levels on the first day of menstruation compared with controls [13]. More research is needed to determine why some individuals suffer more severely.

Current treatment options for menstrual-cycle-related challenges have limitations. The Royal College of Obstetricians and Gynaecologists guidelines recommend hormonal contraceptives or selective serotonin-reuptake inhibitors (SSRIs) as first-line interventions [14]. However, a 2024 meta-analysis found that 39% of individuals with PMS or PMDD failed to respond to SSRIs, and they are associated with side effects including nausea and low libido [15]. Hormonal contraceptives were effective in reducing overall premenstrual symptoms but not depressive premenstrual symptoms specifically, with a 25% dropout rate in trials, potentially indicating poor tolerability [16]. Second-line gonadotrophin-releasing hormone (GnRH) analogs require careful monitoring for bone density loss and menopausal side effects, and a 2025 meta-analysis found insufficient evidence that GnRH analogs with add-back hormone therapy can mitigate these side effects without compromising their global efficacy for PMS and PMDD [17]. For period pain, National Health Service guidance suggests heat or analgesics; however, these are reactive measures that attempt to provide relief only once symptoms have begun affecting the individual [18,19].

Given the limitations of existing approaches, there is interest in nutritional strategies that may proactively support physical and mental wellbeing through the menstrual cycle. There is growing research assessing the effects of certain nutrients and extracts in populations with premenstrual disorders and period pain [[20], [21], [22], [23], [24]]. For example, studies have found that saffron extract and chamomile significantly reduced mood symptoms in PMDD or PMS compared with placebo, with proposed mechanisms involving the serotonergic and GABAergic systems, respectively [[24], [25], [26], [27]]. Meta-analyses found that vitamin D or zinc supplementation reduced the severity of dysmenorrhea compared with placebo, which may relate to effects on prostaglandins and oxidative stress [28,29].

However, individual nutrients typically demonstrate significant effects on some menstrual cycle outcomes but not others. The multifactorial nature and interindividual variability of the menstrual cycle experience suggest that a comprehensive multinutrient formulation may provide a broader supportive approach [8,13]. An important consideration when combining multiple nutrients is the delivery format. Simultaneous release from powder capsules may permit competition between certain nutrients for shared intestinal transporters and compromise absorption [30,31]. To address this, the current formulation employs an advanced “beadlet-in-oil” technology, enabling controlled release to minimize the potential for competitive inhibition [32,33].

This pilot study assessed self-reported changes in premenstrual and menstrual symptoms in females taking a multinutrient supplement containing saffron extract, chamomile extract, chromium, magnesium, zinc, iron, vitamins D, B6, C, and ω–3 (n–3) fatty acids, encapsulated in beadlet-in-oil technology.

## Methods

### Study design

This study was a single-arm, open-label pilot clinical trial to assess changes in self-reported premenstrual and menstrual symptoms in females taking a novel multinutrient supplement. Participants consumed the supplement daily over 3 consecutive menstrual cycles. Questionnaires were completed at baseline and after each menstrual cycle (cycles 1, 2, and 3). Three menstrual cycles were chosen, as previous similar studies are typically 2 to 3 cycles in duration and demonstrate a significant impact of nutrient or extract supplementation compared with placebo, suggesting that this is sufficient time to observe potential effects [[20], [21], [22], [23], 26, [34], [35], [36], [37], [38], [39]].

The study was conducted in accordance with the International Conference on Harmonization Good Clinical Practice guidelines and the United States Code of Federal Regulations applicable to clinical studies (45 CFR Part 46). Ethical approval was granted by the Elemental Institutional Review Board (IRB) (ref: 20736). The study design, execution, and analysis were completed independently of the funder by Citruslabs to eliminate potential bias. The procedures of the trial were documented in a protocol reviewed by the IRB before commencement (7 February, 2025). The study was registered on clinicaltrials.gov (NCT07267728).

### Participants

Participants were recruited via Citruslabs’ proprietary research volunteer database, which consists of individuals who have previously opted in to be contacted for clinical research opportunities. Database members who were female and aged 18 to 45 y (*n* = 12,222) were invited to complete an IRB-approved online screening questionnaire to assess eligibility based on the study’s inclusion and exclusion criteria. Enrollment was completed on a rolling basis. Once the target of 40 eligible participants was reached, no further screening questionnaires were accepted. Of those invited, 211 responded and completed the screening questionnaire before the enrollment target was met. All participants reported concerns related to symptoms associated with their menstrual cycle. Briefly, eligible participants were females aged 18 to 45 y who reported ≥3 menstrual cycle-related symptoms over each of their previous 3 cycles. All participants were eumenorrheic or on hormonal contraception with monthly withdrawal bleeds. Participants were excluded if they had uncontrolled chronic disease, pre-existing chronic conditions that would prevent adherence to the protocol, cardiovascular conditions, polycystic ovary syndrome, hormone-related or reproductive cancers, history of substance abuse, were pregnant or breastfeeding, trying to conceive, or planning surgery during the study period. Only individuals who met all eligibility requirements and provided electronic informed consent were enrolled in the study.

Participants who became inactive (did not complete the questionnaires) at a given timepoint were excluded from the analysis thereafter. Four participants became inactive at cycle 1, and a further 2 at cycle 3, with 36 participants contributing data at cycles 1 and 2, and 34 at cycle 3.

### Intervention

The intervention was the novel multinutrient supplement (2 capsules/d) (supplied by Ditto Daily Ltd), providing a standardized formulation containing saffron extract (*Crocus sativus* L. stigma), chamomile extract (*Matricaria chamomilla* L.), ascorbic acid (vitamin C), chromium picolinate, cholecalciferol (vitamin D_3_), pyridoxal-5-phosphate (P5P; active form of vitamin B_6_), ferrous bisglycinate (iron), magnesium bisglycinate, zinc citrate, and algal oil-derived ω–3 fatty acids (DHA and EPA). Ingredients were selected based on evidence from randomized controlled trials (RCTs) demonstrating effects of nutrient or extract supplements in populations with premenstrual disorders or dysmenorrhea [20,21,23,24,[26], [27], [28], [29],[34], [35], [36], [37],[40], [41], [42]], and vitamin C was included to aid iron absorption [43]. The ingredient composition and dosages are presented in Table 1. Participants took 2 capsules, once daily, in the morning with food and water.

**TABLE 1:** Ingredient composition of the study product (2 capsules)

Ingredient	Dosage/d
Saffron extract (*Crocus sativus* L. stigma)	30 mg
Chamomile oil (*Matricaria chamomilla* L.)	110 mg
Chromium (chromium picolinate)	100 μg
Magnesium (magnesium bisglycinate)	83 mg
Zinc (zinc citrate)	5 mg
Iron (ferrous bisglycinate)	14 mg
Vitamin D3 oil (cholecalciferol)	15 μg (600 IU)
Vitamin B6 (pyridoxal-5-phosphate)	1.4 mg
Vitamin C (ascorbic acid)	80 mg
ω-3 DHA (algal oil-derived)	100 mg
ω-3 EPA (algal oil-derived)	40 mg

Abbreviation: IU, international units.

The ingredients were encapsulated using beadlet-in-oil nutrient delivery technology to minimize nutrient interactions. Chamomile, cholecalciferol, and ω–3 fatty acids were incorporated in oil form, whereas the remaining ingredients comprised dry-form beadlets. The beadlet technology is designed to provide controlled release of nutrients, minimizing competition for absorption pathways [[30], [31], [32], [33]].

### Outcome assessments

The primary outcome was menstrual cycle symptoms assessed using the validated Menstrual Symptom Questionnaire (MSQ). This measures how often psychological and physical manifestations occur for the individual before and during menstruation, assessed on a 5-point Likert scale (1 = never, 2 = rarely, 3 = sometimes, 4 = often, 5 = always) with higher scores indicating more frequent symptoms [44].

The secondary outcome was the severity of 13 individual menstrual cycle symptoms assessed using a 5-point rating scale (0 = not noticeable, 1 = barely noticeable, 2 = moderate, 3 = distracting, 4 = severe). The questionnaire included 6 psychological symptoms (mood swings, irritability, low mood, anxiety, crying spells, difficulty concentrating) and 7 physical symptoms (painful cramps, bloating, fatigue, body aches, breast tenderness, headaches, food cravings).

The questionnaires were completed at baseline and after each menstrual cycle (cycle 1, cycle 2, and cycle 3), each 2 d after the participants’ menstrual bleed to recall their most recent menstrual and luteal phase.

At the end of the study, participants also completed an assessment evaluating their perceived benefit from the supplement. Participants rated their level of agreement with statements regarding symptom improvement (e.g., “The test product reduces my menstrual cramps”), for which they could “Strongly Agree,” “Agree,” “Neither Agree nor disagree,” “Disagree,” or “Strongly disagree.” Participants were also asked about any unexpected reactions or adverse events during the study via an open-ended question.

### Data analysis and statistics

Data were analyzed using R statistical software (R version 4.5.1) and GraphPad Prism (Version 10.6.1). Descriptive statistics were performed on MSQ score and severity data sets to yield means and SDs, which are reported in this study. Following this, a linear mixed-effects model was employed using the study stage as the fixed effect to account for repeated measures within participants, using baseline as the reference level. Post hoc comparisons were conducted using Dunnett’s test for multiple comparisons with baseline as the reference. *P* < 0.05 was considered statistically significant. The linear mixed-effects model and post hoc analysis were applied to each item in the severity questionnaire and overall, whereas MSQ post hoc analysis was performed only on the overall mean score. Percentage changes from baseline were calculated using observed means.

## Results

### Participant characteristics

A total of 40 females were enrolled. The mean age of participants was 33.85 ± 4.19 y (range: 24–41 y), and baseline mean symptom severity scores ranged from moderate to severe. Of these participants, 82.5% were naturally cycling, whereas 17.5% were using hormonal contraceptives with regular monthly withdrawal bleeds.

### Menstrual cycle symptoms

Overall, 88% of participants agreed that the supplement reduced their symptom severity and 82% of participants agreed that the supplement reduced their symptom duration. The MSQ scores decreased significantly and progressively from baseline to cycle 1 (*P* = 0.0028), cycle 2 (*P* < 0.0001), and cycle 3 (*P* < 0.0001) (Table 2).

**TABLE 2:** Summary of changes in premenstrual and menstrual symptoms at baseline, cycle 1, cycle 2, and cycle 3

	Baseline (*N* = 40)	Cycle 1 (*N* = 36)		Cycle 2 (*N* = 36)		Cycle 3 (*N* = 34)	
	Mean ± SD	Mean ± SD	Change (%)	Mean ± SD	Change (%)	Mean ± SD	Change (%)
MSQ score	3.44 ± 0.59	3.13 ± 0.79∗∗	−9.05	2.86 ± 0.81∗∗∗∗	−16.69	2.82 ± 0.79∗∗∗∗	−18.03
Severity scores							
Overall severity	2.58 ± 0.65	1.69 ± 0.82∗∗∗∗	−34.48	1.42 ± 0.78∗∗∗∗	−44.98	1.28 ± 0.83∗∗∗∗	−50.42
Tiredness or fatigue	2.95 ± 0.78	2.17 ± 0.97∗∗∗	−26.55	1.64 ± 1.07∗∗∗∗	−44.44	1.59 ± 1.05∗∗∗∗	−46.16
Bloating	2.58 ± 0.93	2.00 ± 1.22∗	−22.33	1.56 ± 1.03∗∗∗	−39.59	1.44 ± 1.08∗∗∗∗	−44.03
Breast tenderness	2.30 ± 1.04	1.36 ± 1.10∗∗∗∗	−40.82	1.06 ± 0.98∗∗∗∗	−54.11	1.09 ± 0.93∗∗∗∗	−52.69
Headaches	2.35 ± 1.00	1.67 ± 1.10∗∗	−29.08	1.17 ± 0.85∗∗∗∗	−50.35	1.12 ± 1.07∗∗∗∗	−52.44
Painful cramps	2.95 ± 0.78	1.97 ± 1.00∗∗∗∗	−33.15	1.83 ± 1.08∗∗∗∗	−37.85	1.44 ± 0.96∗∗∗∗	−51.15
Body aches	2.58 ± 0.87	1.75 ± 1.00∗∗∗	−32.04	1.44 ± 1.08∗∗∗∗	−43.91	1.15 ± 1.08∗∗∗∗	−55.45
Food cravings	2.73 ± 0.82	1.89 ± 1.19∗∗	−30.68	1.28 ± 1.16∗∗∗∗	−53.11	1.29 ± 1.12∗∗∗∗	−52.51
Feelings of anxiety	2.65 ± 0.86	1.67 ± 1.10∗∗∗∗	−37.11	1.50 ± 1.03∗∗∗∗	−43.40	1.44 ± 1.28∗∗∗∗	−45.62
Irritability	2.98 ± 0.86	1.75 ± 1.03∗∗∗∗	−41.18	1.53 ± 1.03∗∗∗∗	−48.65	1.53 ± 1.08∗∗∗∗	−48.59
Mood swings	2.78 ± 0.95	1.69 ± 1.06∗∗∗∗	−38.94	1.39 ± 0.96∗∗∗∗	−49.95	1.32 ± 1.12∗∗∗∗	−52.31
Crying spells	1.95 ± 1.15	1.08 ± 1.25∗∗	−44.44	1.14 ± 1.18∗∗	−41.60	0.97 ± 1.11∗∗	−50.23
Difficulty in concentration	2.23 ± 0.86	1.39 ± 1.10∗∗∗	−37.58	1.47 ± 1.13∗∗	−33.83	1.03 ± 1.11∗∗∗∗	−53.73
Low mood	2.58 ± 0.98	1.61 ± 1.10∗∗∗	−37.43	1.47 ± 1.11∗∗∗	−42.83	1.24 ± 1.02∗∗∗∗	−52.03

Percentage change was calculated from baseline. ∗*P* < 0.05. ∗∗*P* < 0.01. ∗∗∗*P* < 0.001. ∗∗∗∗*P* < 0.0001. *P* values were obtained from linear mixed-effects models and adjusted using Dunnett’s comparisons.

Abbreviation: MSQ, Menstrual Symptom Questionnaire.

**FIGURE 1:**
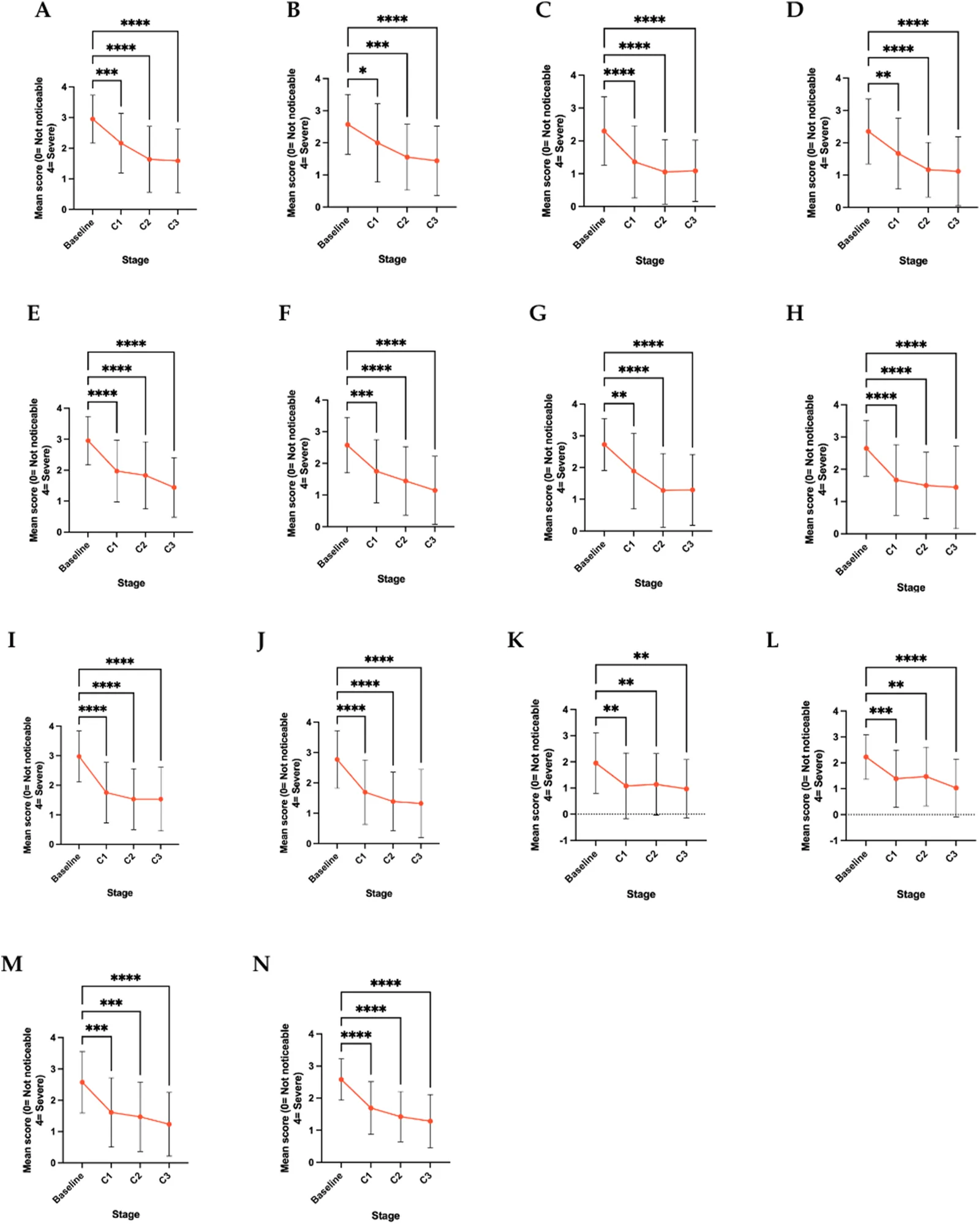
The severity of individual and overall premenstrual and menstrual symptoms at baseline, cycle 1, cycle 2, and cycle 3. (A) Tiredness and fatigue. (B) Bloating. (C) Breast tenderness. (D) Headaches. (E) Painful cramps. (F) Body aches. (G) Food cravings. (H) Feelings of anxiety. (I) Irritability. (J) Mood swings. (K) Crying spells. (L) Difficulty in concentration. (M) Low mood. (N) Overall symptom severity. C1, cycle 1. C2, cycle 2. C3, cycle 3. ∗*P* < 0.05. ∗∗*P* < 0.01. ∗∗∗*P* < 0.001. ∗∗∗∗*P* < 0.0001. *P* values are the result of a linear mixed-effects model using Dunnett-adjusted comparisons to baseline.

Menstrual cycle symptom severity scores demonstrated a significant and progressive decrease over the 3 cycles of taking the intervention (Figure 1, Table 2). Compared with baseline (2.58 ± 0.65), overall severity scores decreased by 34.48% after cycle 1 (1.69 ± 0.82, *P* < 0.0001), 44.98% after cycle 2 (1.42 ± 0.78, *P* < 0.0001), and 50.42% after cycle 3 (1.28 ± 0.83, *P* < 0.0001). This reduction represented a change in symptom severity from “distracting” (between moderate and severe) at baseline to “barely noticeable” by cycle 3.

#### Physical symptoms

All physical symptoms showed significant improvements at cycle 1 (all *P* < 0.02), with continued reductions continuing through to cycle 3 (all *P* < 0.0001) (Figure 1, Table 2). By cycle 3, there were significant reductions from baseline in tiredness or fatigue (−46.16%, *P* < 0.0001), bloating (−44.03%, *P* < 0.0001), breast tenderness (−52.69%, *P* < 0.0001), headaches (−52.44%, *P* < 0.0001), painful cramps (−51.15%, *P* < 0.0001), body aches (−55.45%, *P* < 0.0001), and food cravings (−52.51%, *P* < 0.0001).

#### Psychological symptoms

Similarly, all psychological symptoms showed significant improvements at cycle 1 (all *P* ≤ 0.004), with continued reductions to cycle 3 (all *P* ≤ 0.0028) (Figure 1, Table 2). By cycle 3, there were significant reductions from baseline in feelings of anxiety (−45.62%, *P* < 0.0001), irritability (−48.59%, *P* < 0.0001), mood swings (−52.31%, *P* < 0.0001), crying spells (−50.23%, *P* = 0.0028), difficulty concentrating (−53.73%, *P* < 0.0001), and low mood (−52.03%, *P* < 0.0001).

### Participant perceptions and satisfaction

At the end of the study, the majority of participants reported perceived benefits for reduced cramping (91% of participants), improved mood (82% of participants), fewer mood changes (88% of participants), reduced bloating (71% of participants), reduced breast tenderness (71% of participants), and fewer headaches or migraines (76% of participants).

Participants reported high satisfaction and intent to continue use, with 94% agreeing they would like to continue taking the supplement after the trial and 91% agreeing they would recommend it to friends and family.

### Adverse effects

One participant reported that they noticed crying spells during cycle 3, whereas they had improvement in other symptoms.

## Discussion

This pilot study aimed to assess self-reported changes in premenstrual and menstrual symptoms among females taking a novel multinutrient supplement and found perceived progressive decreases across 13 physical and psychological symptoms over 3 menstrual cycles. These changes were consistent across all measured symptoms, suggesting they were perceived broadly across the menstrual cycle symptom spectrum. Notably, participant satisfaction was high, with 94% expressing that they would like to continue supplementation beyond the trial period. The preliminary findings of the current study suggest that the supplement may be of interest as a nutritional approach for individuals experiencing menstrual cycle-related symptoms, warranting confirmation in a randomized, placebo-controlled trial.

A high proportion (88%) of participants agreed that they felt reduction in the severity of overall symptom in this study. Further to this, the magnitude of change in severity scores was −50% (range: −44% to −55%), exceeding a clinically meaningful threshold of 43% previously reported by Borenstein et al. [45] that has been associated with significant differences in quality of life, work productivity, and healthcare costs. This is especially important as females with premenstrual disorders are found to have lower quality of life and relationship satisfaction, a 3.83- to 5.32-fold higher risk of absenteeism, 40% higher risk of sick leave, and 27% higher risk of unemployment [4,46,47]. Future studies evaluating the supplement should include validated quality of life measures to assess whether self-reported symptom change translates into real-world functional outcomes.

The perceived temporal burden of menstrual cycle symptoms also decreased across the study, as 82% of participants agreed they felt reduced symptom duration, and MSQ scores declined by 18%. This suggests that the supplement may be associated with changes in not only how severe symptoms feel but also how long they last and how often they occur.

A progressive decrease in symptoms across each menstrual cycle was observed, with the largest change after 3 cycles. A similar pattern of progressive reductions over successive cycles has been reported in previous RCTs of individual ingredients, including saffron extract, ω–3, and magnesium [22,26,37,40]. This is consistent with the broader principle that nutritional interventions often require sustained exposure before physiological effects become measurable [48,49]. Future studies with longer follow-up (e.g., 6 menstrual cycles) and biomarker measurements may help establish whether perceived improvement continues beyond 3 cycles and whether it is associated with underlying physiological changes over time.

Existing literature offers insight into plausible mechanisms by which nutrients may influence the outcomes assessed in this study. Vitamin D, zinc, ω–3 fatty acids, and magnesium have each shown significant effects on dysmenorrhea in placebo-controlled trials [28,29,34,[40], [41], [42], [43], [44], [45], [46], [47], [48], [49], [50]]. Separately, mechanistic studies find that vitamin D may modulate prostaglandins through regulatory action on enzymes involved in both prostaglandin synthesis (cyclooxygenase-2) and degradation (15-hydroxyprostaglandin dehydrogenase) [[51], [52], [53], [54]]. Zinc has been found to function as an antioxidant, and 1 study found that zinc supplementation was associated with increased total antioxidant capacity and reduced premenstrual symptoms [12,35]. Magnesium may prevent excessive muscle cell depolarization by competitively inhibiting calcium ion entry through voltage-gated calcium channels at the cell membrane, thereby reducing excessive contractions. However, this mechanism is suggested from a study of its role in labor and not confirmed for menstrual cramps specifically [34,55].

For mood symptoms, saffron extract has shown benefits for PMS and PMDD symptoms in placebo-controlled trials, and it is proposed that this may be partly due to its compounds, safranal and crocin, influencing neurotransmitter systems, particularly by inhibiting serotonin reuptake, a mechanism identified in rodent behavioral and neurochemical studies [[24], [25], [26]]. Chamomile extract significantly reduced mood symptoms compared with placebo in those with menstrual-related mood disorders [21]. Chamomile contains apigenin, a compound that has been shown in in vitro models to potentially cross the blood–brain barrier, and which is proposed in mechanistic animal studies to interact with GABA and N-methyl-D-aspartate receptors to promote mood-stabilizing and anxiolytic effects [27,56]. These potential mechanisms are particularly relevant given that serotonergic and GABAergic system dysregulation has been documented in PMDD and PMS [9,10,57]. Further to this, zinc supplementation increased brain-derived neurotrophic factor (BDNF) in participants for whom it improved PMS [35], and BDNF is suggested to support neurogenesis and the function of serotonergic neurons based on findings from animal studies [58]. Each of these mechanisms represents a biologically plausible pathway through which the supplement’s component ingredients could support menstrual wellbeing, and each should be considered a candidate when deciding which biomarkers to assess in future controlled trials.

Beyond premenstrual symptoms, several ingredients within the novel multinutrient supplement have demonstrated potential for other areas of females’ health. For example, chamomile extract has been shown to reduce pelvic pain, dysmenorrhea, and dyspareunia in patients with endometriosis and alleviate symptoms of cyclic mastalgia [36,59]. Furthermore, saffron extract was associated with greater improvements in psychological symptoms in perimenopausal females [60]. The interest in this supplement has the potential to extend across multiple underserved areas of females’ health, and future research should evaluate this.

Safety considerations were paramount in formulating the novel multinutrient supplement, particularly regarding vitamin B6 dosing. The Royal College of Obstetricians and Gynaecologists guidelines suggest vitamin B6 as a simple measure for the management of premenstrual symptoms, while cautioning that studies typically use higher doses that may induce peripheral neuropathy [14]. The United Kingdom Department of Health restricts the daily supplemental dose of vitamin B6 to 10 mg because of neurotoxicity concerns, yet many commercially available supplements contain doses exceeding this safety threshold. Accordingly, the novel multinutrient supplement formulation adheres to the safety limit, balancing therapeutic benefit with patient safety and ensuring long-term tolerability for daily use.

Strengths of this study include its 3-cycle duration, allowing observation of change over time and providing valuable data to inform expectations. This study intentionally used a broad inclusion criterion to capture the diverse range of premenstrual and menstrual symptom presentations. Participants were included if they had concerns relating to 3 or more menstrual cycle symptoms, with a mean severity of moderate to severe at baseline. The study’s inclusive design also permitted enrollment of participants using hormonal contraception, as many individuals take hormonal contraceptives yet continue to experience symptoms. The 94% continuation intent rate is a promising indicator of acceptability and feasibility of daily supplementation in real-world settings. Limitations of this study include the lack of a control group, limiting causal inference compared with that of an RCT, which is required to confirm these findings. Furthermore, adherence was not measured, so it is not certain whether participants took the supplement as directed, and any variation could have influenced the symptom changes reported.

This pilot clinical trial found that participants self-reported reduced premenstrual and menstrual symptoms over 3 menstrual cycles of daily supplementation. The magnitude of change observed was notable, with symptoms shifting from “distracting” at baseline to “barely noticeable” by the end of the study, which may be of relevance to daily functioning and quality of life; however, this was not directly assessed. An RCT of the same duration in 90 females is now underway to confirm these preliminary findings and includes a placebo control, adherence monitoring, additional validated symptom questionnaires, and quality of life measures; a longer follow-up period remains a consideration for subsequent studies.

## Author contributions

The authors’ responsibilities were as follows – AvdS, SM: designed research; SM: conducted research; AvdS: provided essential reagents or provided essential materials and primary responsible for final content; SM: analyzed data or performed statistical analysis; MS, AvdS: wrote the paper; and all authors: read and approved the final manuscript.

## Data availability

Data described in the manuscript will be made available on request pending application and approval by the corresponding author.

## Declaration of Generative AI and AI-assisted technologies in the writing process

The authors declare that no generative AI or AI-assisted technologies were used in the writing of this manuscript.

## Funding

This research was funded by Ditto Daily Ltd.

## Conflict of interest

MS and AvdS are employees of Ditto Daily Ltd, who funded this study and supplied the supplement consumed by participants in the trial, but had no role in study conduct, data collection, or analysis. Citruslabs was commissioned by Ditto Daily Ltd to independently complete this study.
